# Advanced FCS-MPC strategy for optimized control and efficiency in photovoltaic inverters

**DOI:** 10.1038/s41598-026-39371-0

**Published:** 2026-02-19

**Authors:** A. Dekhane, A. Djellad, Maissa Farhat, A. Khouzam, P. O. Logerais, Maen Takruri, Aws Al-Qaisi

**Affiliations:** 1 National Higher School of Technology and Engineering, Department of Electronics, Electrical Engineering and Automation, Annaba, 23005 Algeria; 2 School of Engineering, American University of Ras Al Khaimah, Building: 75, Seih Al Araibi, Ras Al Khaimah, United Arab Emirates; 3 Univ Paris Est Créteil, CERTES, 61 avenue du Général de Gaulle, F-94010 Créteil Paris, France; 4 ICAM Grand-Paris Sud, Carré Sénart, 34 Points de Vue, Lieusaint, 77127 France; 5 College of Engineering and Technology, American University of the Middle East, Egaila, 54200 Kuwait; 6 Environmental Research Center (CRE), Annaba, Algeria

**Keywords:** FCS-MPC, Predictive control, PV inverter, Harmonic distortion, Efficiency, Grid integration, Two-step prediction, Voltage sag, Energy science and technology, Engineering

## Abstract

This paper proposes an enhanced Finite Control Set Model Predictive Control (FCS-MPC) approach to optimize the performance and efficiency of grid-connected photovoltaic (PV) systems. The novelty of this study lies in applying a two-step forward prediction scheme within the FCS-MPC framework, coupled with optimized cost functions, to improve control accuracy, harmonic reduction, and transient response. A 1 MW industrial-scale PV system model, based on the Oued El Kebrit power plant in Algeria, is simulated to evaluate the controller under realistic grid disturbances. Simulation results demonstrate that the proposed strategy improves efficiency from 97.63% to 97.73%, reduces voltage Total Harmonic Distortion (THDv) to 2.08%, and shortens the voltage stabilization time from 0.25s to 0.165s. Furthermore, the method ensures consistent performance during grid faults such as voltage sags and maintains grid code compliance. The proposed FCS-MPC method outperforms conventional strategies, offering a scalable and robust solution for enhancing the energy conversion and stability of large-scale PV systems.

## Introduction

The urgency to transition toward sustainable energy sources has highlighted the importance of photovoltaic (PV) systems as a cornerstone of the global energy mix^1,2^. These systems offer a viable solution to reducing reliance on traditional power sources by transforming solar energy into electricity, which can supplement the grid or meet local energy needs, promoting energy independence and contributing to a more sustainable future^3^. As the demand for renewable energy increases, PV systems have become crucial in distributed generation^4^. These systems are mainly categorized into standalone (off-grid) and grid-connected (on-grid) systems, the latter gaining prominence due to its ability to feed excess power back into the grid, enhancing scalability and matching energy demands^5^. The widespread adoption of grid-connected PV systems also supports economic development by creating new employment opportunities^6^.

A typical grid-connected solar PV system consists of solar panels, PV arrays, Maximum Power Point Tracking (MPPT) systems^7^, filters, DC-DC converters, and inverters^8^. In particular, the inverter plays a vital role in converting direct current (DC) from PV panels into alternating current (AC) compatible with the grid^9^. However, integrating these systems with the power grid presents challenges beyond simple power conversion^10^. To effectively incorporate PV systems into the grid, advanced control strategies are required to address the variability and intermittency of solar power while ensuring grid stability and compliance with quality standards^11^. These control strategies are highly expected to optimize the performance of the PV system under varying solar conditions while ensuring compliance with the grid regulations^12^.

Two-level inverters are recognized for their reliability and cost-effectiveness in grid-connected PV applications among the various inverter topologies^13^. Unlike multilevel inverters, which incorporate multiple voltage levels and more complex designs, two-level inverters switch between two voltage levels, typically + V_dc_ and -V_dc_, to generate a sinusoidal AC waveform. The simplicity of this design minimizes the costs of the system and improves the operational reliability^14^. However, as the demand for higher efficiency and improved power quality grows, multilevel inverters, which theoretically offer reduced harmonic distortion and better voltage control, have become more common in the literature and in the industry^15^. Despite their advantages, two-level inverters remain a practical and widely used solution, especially in small to medium scale PV systems, providing high efficiency (often exceeding 95%) and being well-understood in design and implementation^16,17^. While multilevel inverters help reduce harmonic distortion, they introduce challenges such as higher component costs, increased installation complexity, and higher electromagnetic interference (EMI), which can negatively impact the performance of the system^18,19^.

The proposed control technique demonstrates how a two-level inverter, when combined with a predictive control framework like FCS-MPC, can serve as a cost-effective and high-performance solution for grid-connected PV systems. Despite the popularity of multilevel inverters, the simplicity and lower component count of two-level inverters make them ideal for scalable deployment, particularly when equipped with advanced control strategies^20^. Recent literature on grid-connected PV systems and inverter control strategies has explored challenges, such as voltage sags and power quality. Inverter topologies, including transformer-based, transformer-less, multilevel, and hybrid systems, each offer unique advantages and challenges regarding efficiency and complexity^21^. Control strategies, such as proportional-integral (PI), proportional-resonant (PR), deadbeat, and predictive controllers^22–24^, play a critical role in maintaining the performance of the system and the grid stability. These strategies are assessed based on their complexity, stability, robustness, and impact on the quality of the power^25^. Moreover, modern inverter technologies incorporate low-voltage ride-through (LVRT) capabilities, which are essential for grid-code compliance and addressing grid faults^25^.

The future of PV systems is closely tied to advancements in inverter technologies, with trends pointing toward cost reduction, higher reliability, and multifunctionality in inverter designs^26^. Emerging materials, such as silicon carbide and gallium nitride, are set to improve the efficiency and longevity of PV systems, which will further support the widespread adoption of renewable energy technologies^27^. Recent advancements in single-phase grid-connected inverters, including non-isolated, isolated, and resonant topologies, highlight the importance of control methods such as current injection and grid-connection techniques for achieving optimal performance of the system^28^.

The control strategies of the inverter require also to consider techno-economic factors, such as balancing performance and cost-effectiveness. Studies on techno-economic optimization underscore the importance of matching inverter sizing according to the power of the PV module and considering the installation costs and payback periods^29^. Predictive model control in inverters, primarily through MPC, improves the dynamic response, the quality of the power, and the efficiency by regulating the output voltage and current^30^. It minimizes the fluctuations of the voltage that could lead to inefficiencies, thermal stress, or potential damage to system components while ensuring stable output and compliance with the standards of the grid^28^. The flexibility of the MPC technique and its adaptability make it an ideal control strategy for mitigating the harmonic distortion and maintaining quality of the power in grid-connected PV systems^31^.

Model Predictive Control (MPC) has increasingly been applied in power converters, particularly in two-level inverters^32^, due to its ability to reduce harmonic distortion and enhance overall power quality^33^. This real-time predictive ability enables MPC to manage dynamic grid conditions effectively, ensuring stable operation, even in challenging scenarios. Studies have demonstrated that the effectiveness of the MPC technique in reducing Total Harmonic Distortion (THD) and improving the efficiency of the system^34,35^. Specifically, in two-level inverters, MPC mitigates the harmonic content typically associated with simpler control strategies, such as Pulse Width Modulation (PWM)^36,37^.

This paper proposes an enhanced inverter control strategy using Finite Control Set Model Predictive Control (FCS-MPC), with a focus on large-scale, grid-connected PV systems. The key contribution lies in implementing a two-step forward prediction mechanism within the FCS-MPC structure, combined with tailored cost functions for both current and power. This allows for improved voltage regulation, reduced harmonic distortion, and faster dynamic response under grid disturbances.

While existing studies have explored FCS-MPC for inverter control, most are limited to small-scale test systems and do not investigate its behavior under real-world fault conditions such as voltage sags or phase jumps. Moreover, many lack comparative evaluation of cost functions or analysis of response delays due to sampling constraints. Our work addresses these gaps through a detailed simulation study based on a 1 MW PV system configuration inspired by the Oued El Kebrit plant in Algeria^35,38–40^.

This distinguishes our work from similar studies, which often focus on smaller laboratory-scale installations and low-power systems, limiting their applicability to large-scale scenarios. The ability to maintain consistent power output, even during grid faults, using the FCS-MPC approach marks a significant advancement over the traditional control strategies. In particular, PI-based or adaptive controllers often struggle to handle dynamic grid conditions at larger scales. Our approach, however, demonstrates robust performance, highlighting its potential for deployment in large-scale PV systems.

The paper is organized as follows: Section II presents the system configuration and control methodology. Section III outlines the FCS-MPC design, including current-based and power-based cost functions. Section IV provides detailed simulation results under varying operating conditions, including grid faults and step-load variations. Finally, Section V concludes with key findings and directions for future research, particularly regarding real-time implementation and computational optimization.

## System and control strategy

**Fig. 1:**
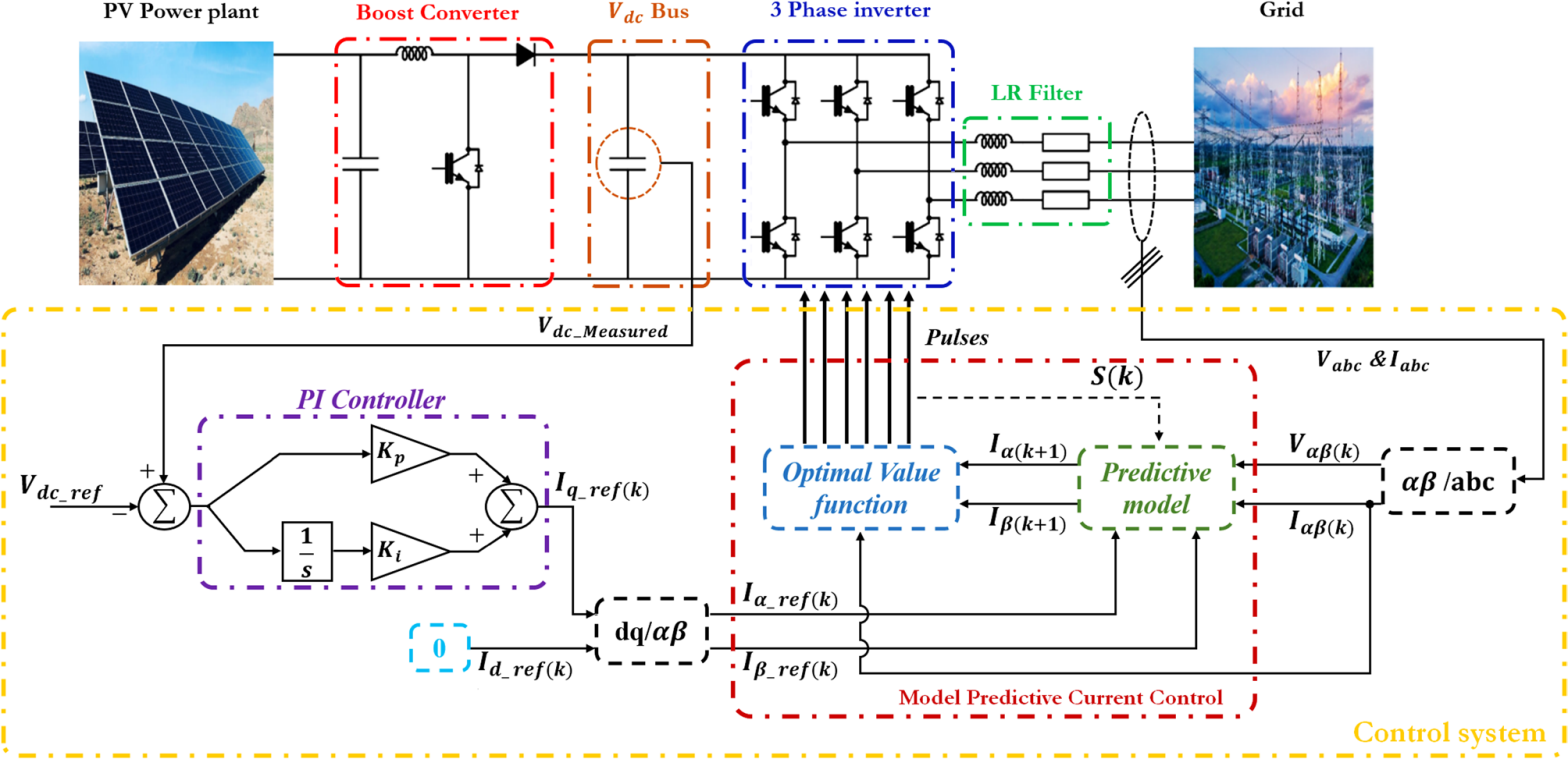
Studied system with the proposed control. Note: $\:{i}_{dref}\left(k\right)$ is set to zero in this configuration to ensure unity power factor and simplify current regulation using dq-axis decoupling.

The considered system depicted in Fig. 1 consists of a three-phase, two-level inverter employing insulated-gate bipolar transistors (IGBTs) that are connected to a DC bus, and stabilized by a capacitor to smooth voltage fluctuations.

The inverter output is filtered through an RL series filter before connecting to the grid, ensuring compliance with the power quality standards by mitigating the harmonics. The DC bus voltage is regulated using a proportional-integral (PI) controller that processes the reference and the measured voltage values. Additionally, the inverter control relies on a Finite Control Set Model Predictive Control (FCS-MPC) algorithm, which dynamically predicts and optimizes the performance of the system under varying grid and operating conditions.

This work uses a self-commutated voltage source inverter, a power electronic converter controlled by voltage levels. IGBT is usually used for 1 MW power, exceeding 100 kW and under 20 kHz low-frequency range. The DC bus voltage remains constant, determining the average power flow direction and shaping the output AC voltage waveform. The DC bus voltage is regulated using a PI controller with two input signals, $\:{V}_{ref}$ and $\:{V}_{meas}$, as depicted in Fig. 1.

FCS-MPC is a control strategy that operates at each sampling interval. It uses a predictive model of the system to evaluate all the possible control actions and selects the one that minimizes a predefined cost function. This process anticipates the future behavior of the system within a finite time horizon, ensuring optimal performance under dynamic operating conditions^41,42^. The FCS-MPC involves a set of switching states for the inverter, contingent on the number of levels and the architecture of the inverter. For this study, a three-phase two-level inverter is employed, and the corresponding switching states are presented in Table 1.

**Table 1:** Switching states^2^.

Voltage vectors	Switching states ($\:{S}_{abc}$)	Output voltages ($\:{v}_{\alpha\:},{v}_{\beta\:}$)		Output voltage ($\:{v}_{a}{,\:v}_{b,},{v}_{c}$)		
$\:{u}_{0}$	000	0	0	0	0	0
$\:{u}_{1}$	100	$\:\frac{{2v}_{dc}}{3}$	0	$\:\frac{{2v}_{dc}}{3}$	$\:\frac{{-v}_{dc}}{3}$	$\:\frac{{-v}_{dc}}{3}$
$\:{u}_{2}$	110	$\:\frac{{v}_{dc}}{3}$	$\:\frac{{\sqrt{3}v}_{dc}}{3}$	$\:\frac{{v}_{dc}}{3}$	$\:\frac{{v}_{dc}}{3}$	$\:\frac{-{2v}_{dc}}{3}$
$\:{u}_{3}$	010	$\:\frac{{-v}_{dc}}{3}$	$\:\frac{{\sqrt{3}v}_{dc}}{3}$	$\:\frac{{-v}_{dc}}{3}$	$\:\frac{{2v}_{dc}}{3}$	$\:\frac{{-v}_{dc}}{3}$
$\:{u}_{4}$	011	$\:\frac{-{2v}_{dc}}{3}$	0	$\:\frac{-{2v}_{dc}}{3}$	$\:\frac{{v}_{dc}}{3}$	$\:\frac{{v}_{dc}}{3}$
$\:{u}_{5}$	001	$\:\frac{{-v}_{dc}}{3}$	$\:\frac{-{\sqrt{3}v}_{dc}}{3}$	$\:\frac{{-v}_{dc}}{3}$	$\:\frac{{-v}_{dc}}{3}$	$\:\frac{{2v}_{dc}}{3}$
$\:{u}_{6}$	101	$\:\frac{{v}_{dc}}{3}$	$\:\frac{-{\sqrt{3}v}_{dc}}{3}$	$\:\frac{{v}_{dc}}{3}$	$\:\frac{-{2v}_{dc}}{3}$	$\:\frac{{v}_{dc}}{3}$
$\:{u}_{7}$	111	0	0	0	0	0

Now that the base of the MPC has been laid, various MPC variants exist depending on the cost function^32^. This work focuses on the power and current cost functions as these two are the only ones that enable the power flow control, facilitating a comparative analysis^43^.

***- Current-based FCS-MPC control***: Several variations exist for the current cost function. Some variations focus solely on current, while others include voltage with weight in attempt to improve the outcome. Other approaches consider predictions at step $\:k+1$, or at steps $\:k+2,\:k+3$, or all the predictions at once. However, considering more steps reduces the calculation speed. Here, we consider only $\:k+1$ and $\:k+2$. The cost function to minimize, based on the one-step prediction ( $\:k+1$), is as follows:

1 $$\:g=\left|{i}_{d}\left(k+1\right)-{i}_{dref}\left(k\right)\right|+\left|{i}_{q}\left(k+1\right)-{i}_{qref}\left(k\right)\right|$$

The new cost function is (two steps prediction $\:k+2$):

2 $$\:g=\left|{i}_{d}\left(k+2\right)-{i}_{dref}(k+1)\right|+\left|{i}_{q}\left(k+2\right)-{i}_{qref}(k+1)\right|$$

In the proposed control strategy, the reference for the direct-axis current component, $\:{i}_{dref}\left(k\right)$, is set to zero to maintain unity power factor. This allows the entire active power to be regulated via the quadrature component (Iq), simplifying the predictive control algorithm.

**Fig. 2:**
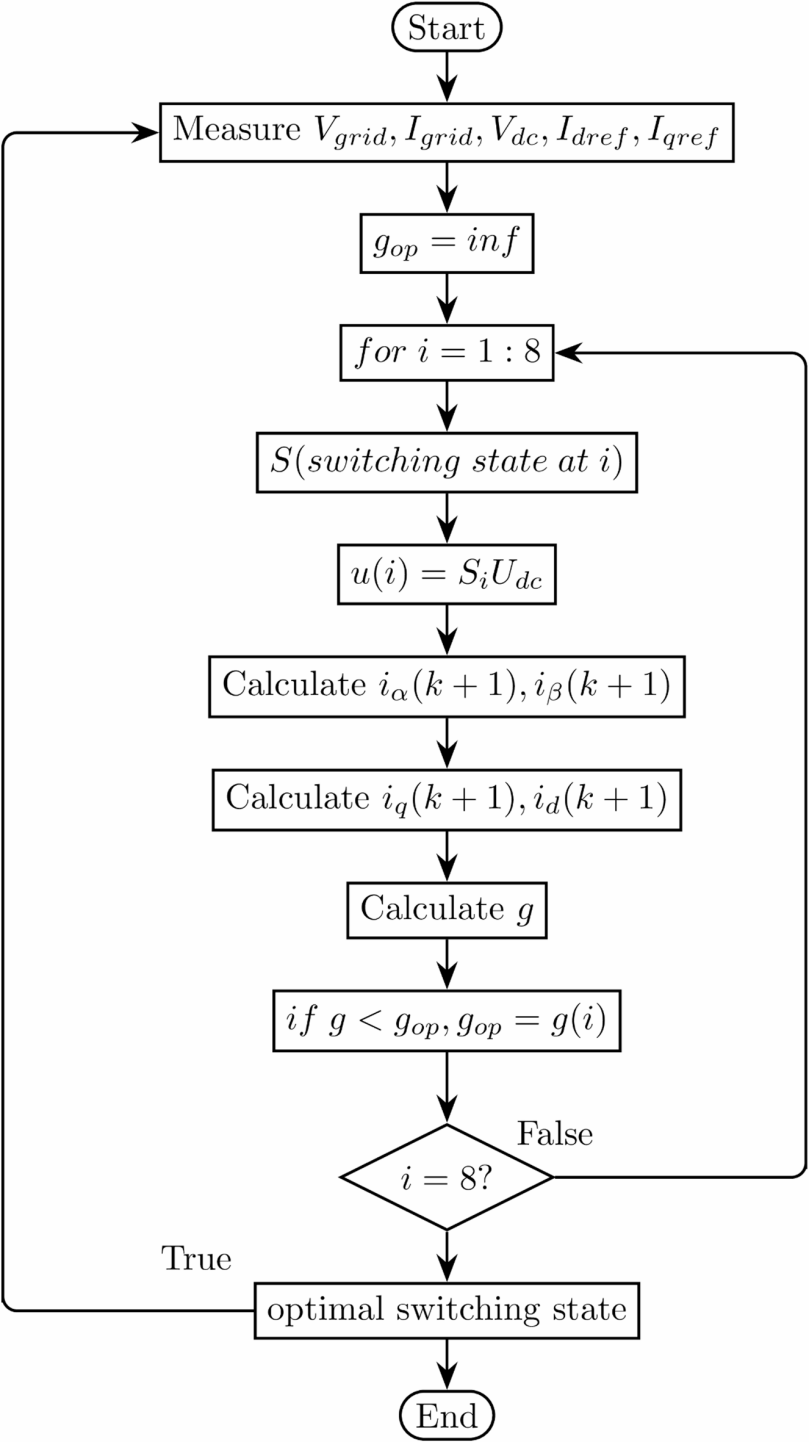
Flowchart of current cost function MPC.

Figure 2 illustrates the flowchart for the Finite Control Set Model Predictive Control (FCS-MPC) process based on the current cost function. The process begins with the state measurements of the system, such as voltage and current, which serve as inputs to the predictive model. The predictive model calculates the potential future states of the system based on all the possible switching actions of the inverter. A cost function evaluates these predicted states, identifying the action that minimizes the deviation from the desired reference values. The optimal switching state is then applied to the inverter and also to update the system at the next sampling interval. This iterative process ensures precise control of the inverter output, minimizing the harmonic distortion and ensuring efficient power flow.

***Power-based FCS-MPC control***: Model predictive control with a power cost function can address grid faults such as voltage sags especially when combined with algorithms such as LVRT, though this aspect will not be explored in this study^44,45^.

The new cost function is (for one-step prediction k + 1):

3 $$\:g={\omega\:}_{p}{\left[P\left(k+1\right)-{P}_{dref}\left(k\right)\right]}^{2}+{\omega\:}_{q}{\left[Q\left(k+1\right)-{Q}_{qref}\left(k\right)\right]}^{2}$$

For the two steps of forward prediction k + 2, the reference power values are obtained through the PI controller for the active power and a constant block is used to control the reference reactive power. The new cost function is:

4 $$\:g={\omega\:}_{p}{\left[P\left(k+2\right)-{P}_{dref}\left(k+1\right)\right]}^{2}+{\omega\:}_{q}{\left[Q\left(k+2\right)-{Q}_{qref}\left(k+1\right)\right]}^{2}$$

## Case study and simulation

**Fig. 3:**
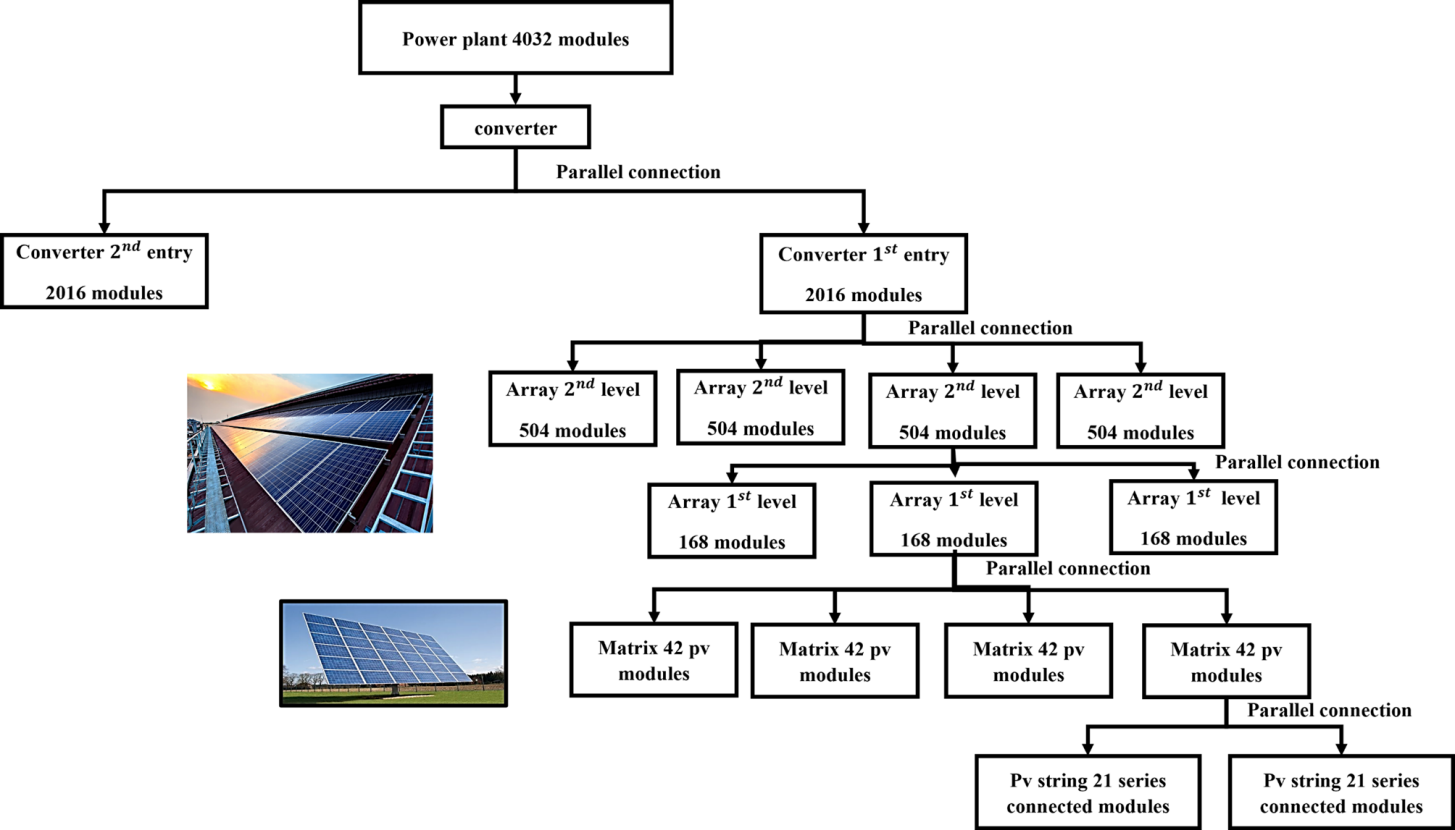
System structuration^46^.

The configuration of the 1 MW photovoltaic (PV) system, comprising 4032 modules, is directly inspired by the Oued El Keberit photovoltaic power plant in the Souk Ahrass region of Algeria. This configuration was selected as it mirrors a real-world, large-scale PV system, providing a relevant foundation for the simulations in this study. The system setup was chosen to reflect typical conditions for industrial-scale PV installations operating in hot climates, where environmental factors such as high temperatures and fluctuating solar irradiance significantly impact the performance of the system.

This facility, with a total capacity of 15 MW, is structured as fifteen identical 1 MW units. Our study isolates one of these units as a representative configuration. This configuration is illustrated in Fig. 3, showing the layout of the system. The detailed characteristics of the modules are detailed in Table 2^46–48^.

**Table 2:** Characteristics of one PV module.

Parameters	Values
*STC Power Rating*	*250 W*
*STC (Power per unit of area)*	*153.0 W/m*^*2*^ *(14.2 W/ft*^*2*^*)*
*Peak Efficiency*	*15.3%*
*Number of Cells*	*60*
*I*_*mp*_	*0.24 A*
*V*_*mp*_	*30.4 V*
*I*_*sc*_	*8.79 A*
*V*_*oc*_	*38.4 V*

While environmental factors such as high temperatures and fluctuating solar irradiance are significant in industrial-scale PV installations, the focus of our study was not on these conditions. Instead, we prioritized evaluating the robustness of the proposed control strategy under dynamic grid scenarios, particularly under grid faults like voltage sags. These grid disturbances, lasting between 100 ms and 500 ms and reaching up to 30% of the nominal voltage, were central to assessing the system’s performance.

The system setup includes a three-phase, two-level inverter connected to the grid through an RL filter, with a regulated DC bus voltage of 800 V. The Total Harmonic Distortion (THD) is maintained below 5% in compliance with IEEE 519 standards, and voltage and current deviations are limited to ± 2% of their reference values. The simulations also account for these grid disturbances to evaluate the robustness of the control strategy. Key variables such as DC bus voltage, grid current, active and reactive power, and voltage waveforms at the Point of Common Coupling (PCC) were focused on to assess the system’s behavior under these dynamic conditions. The input conditions for step size, irradiance, and temperature, although critical for other performance evaluations, were not the primary focus of this study.

## Results and discussion

### Current-based FCS-MPC control

*One-step prediction k + 1*

**Fig. 4:**
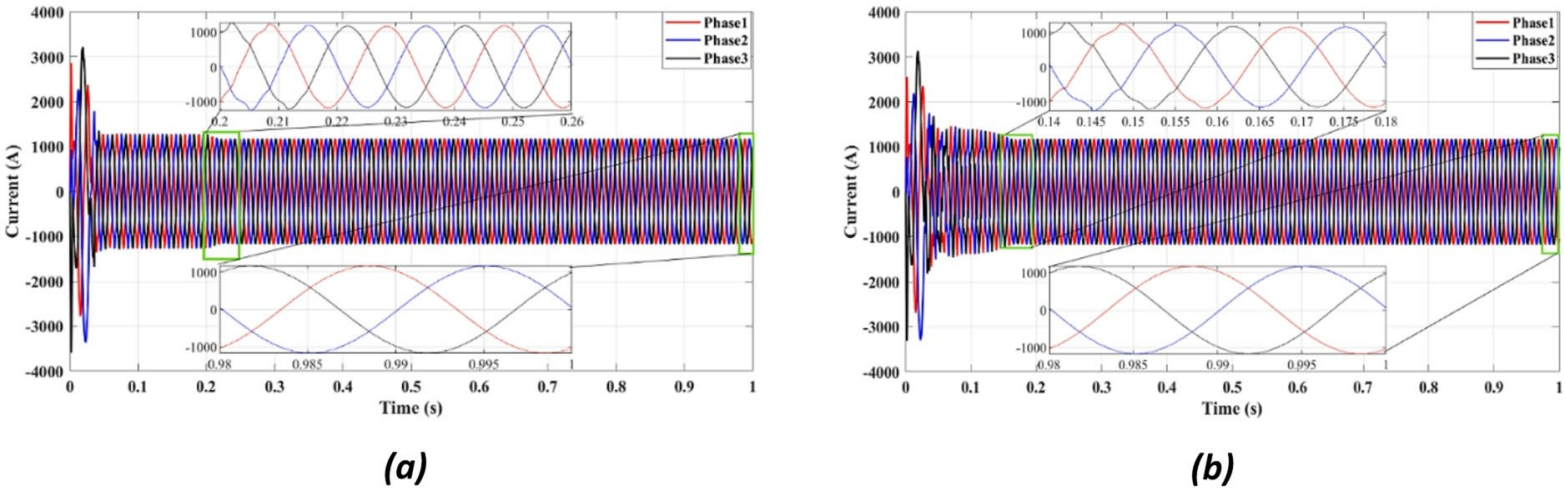
Three-phase grid current waveform at the PCC for (**a**) Absolute value cost function, (**b**) Squared cost function.

**Fig. 5:**
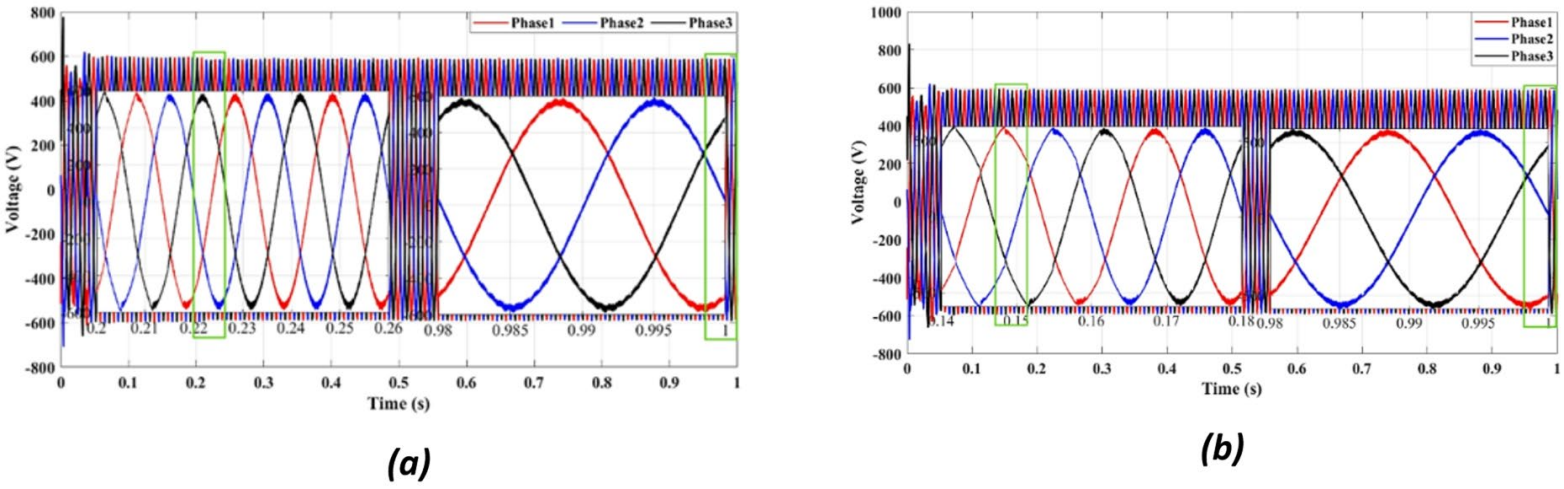
Three-phase grid voltage waveform at the PCC for **(a)** Absolute value cost function, (**b**) Squared cost function.

Figures 4 and 5 present the waveforms of the grid current and voltage across all three phases, measured at the LR filter output identified as the Point of Common Coupling (PCC). The simulation results reveal a transitional phase of approximately 0.25 s before the current and the voltage stabilize for the absolute cost function, as shown in Figs. 4a and 5a. In the case of the squared cost function, the system achieves a steady state after 0.165 s, as depicted in Figs. 4b and 5b.

**Fig. 6:**
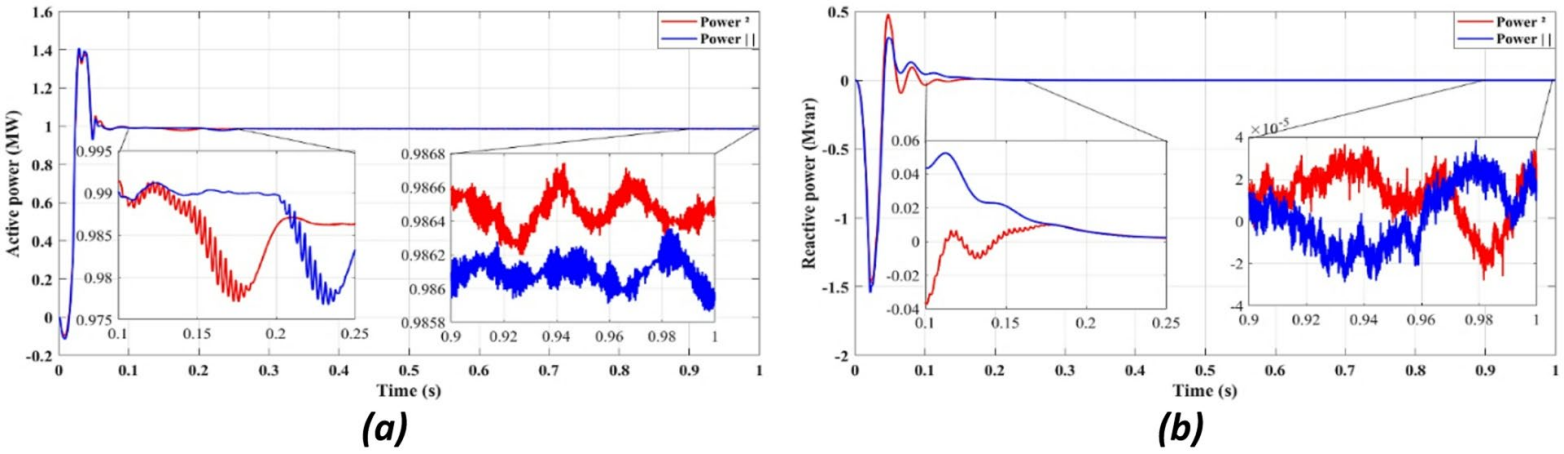
Showcases simulation results of independent control: (**a**) Active power, (**b**) Reactive power.

Figure 4 compares the three-phase grid current waveforms at the PCC using different cost functions. Figure 4b (two-step prediction) shows a clear improvement in response time, with the current stabilizing within 0.165s, compared to 0.25s in Fig. 4a (one-step prediction). This faster stabilization enhances dynamic response, reducing power oscillations and improving the reliability of the system. Figure 5 illustrates the three-phase grid voltage waveform at the PCC. Figure 5b (two-step prediction) shows a faster voltage stabilization, lowering fluctuations compared to Fig. 5a (one-step prediction). The two-step approach reduces the transient period to 0.165s, ensuring improved voltage regulation. These results confirm that the squared cost function leads to a smoother and more stable voltage profile, which is essential for grid compliance and improvement of the power quality. Figure 6a displays the active power output, while Fig. 6b shows reactive power control. The simulation results indicate that the absolute value cost function achieves an efficiency of 97.63% (0.9861 MW out of 1.01 MW), while the squared cost function further improves efficiency to 97.66% (0.9864 MW out of 1.01 MW). Although the difference is small, this 0.03% gain in efficiency may translate to substantial energy savings in the case of a large-scale PV system. Furthermore, Fig. 6b confirms that the reactive power is kept at almost zero, ensuring improved grid stability.

**Fig. 7:**
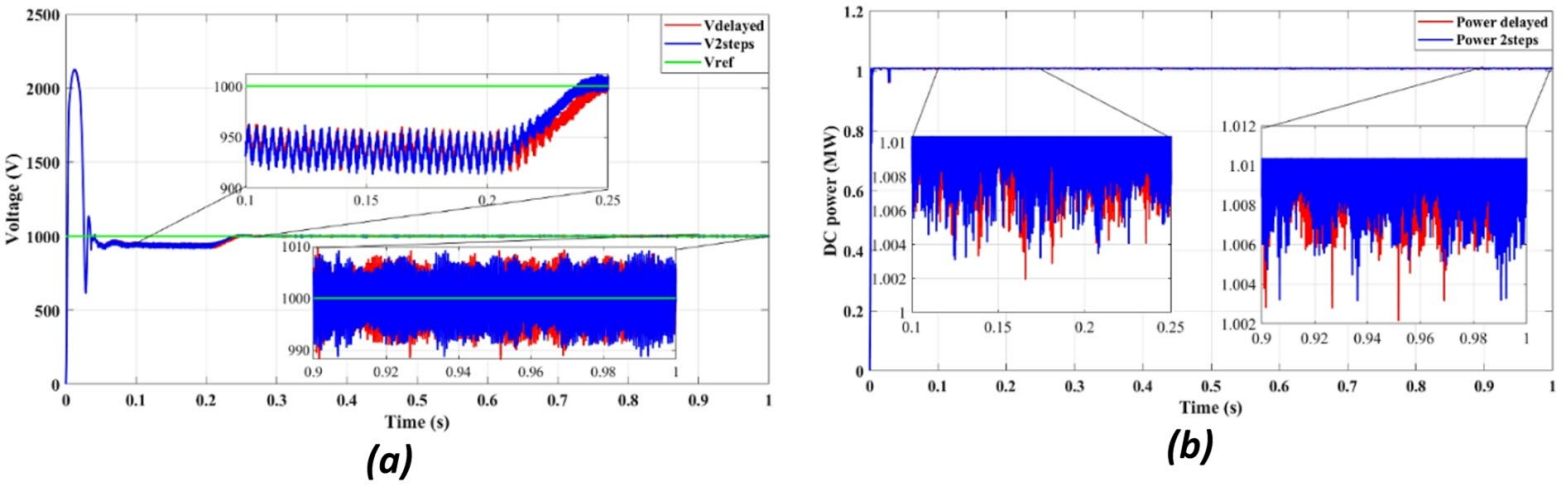
DC bus: (**a**) Voltage, (**b**) Power.

Figure 7a presents DC bus voltage stability, while Fig. 7b shows the power flow behavior. The results confirm that the squared cost function reduces the fluctuations of the transient voltage and reaches a steady state faster than the absolute value of the cost function. As seen in Fig. 7a, the squared cost function achieves a stable voltage level within 0.165s, while the absolute function requires 0.25s. This improvement in the transient response helps to reduce voltage dips, preventing stress of the inverter and enhancing the longevity of the overall system.

*Two steps prediction k + 2*

**Fig. 8:**
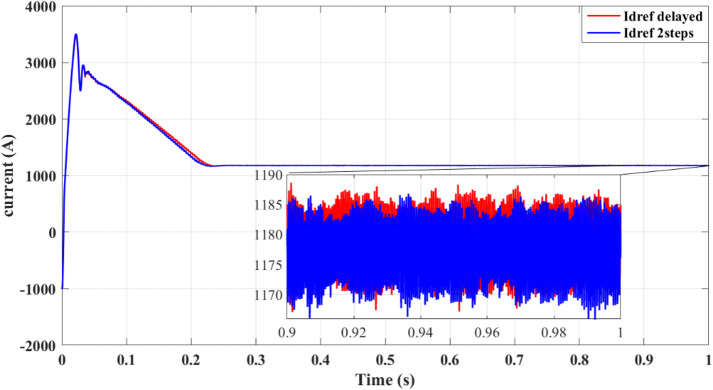
Reference current signal $\:{i}_{dref}$.

**Fig. 9:**
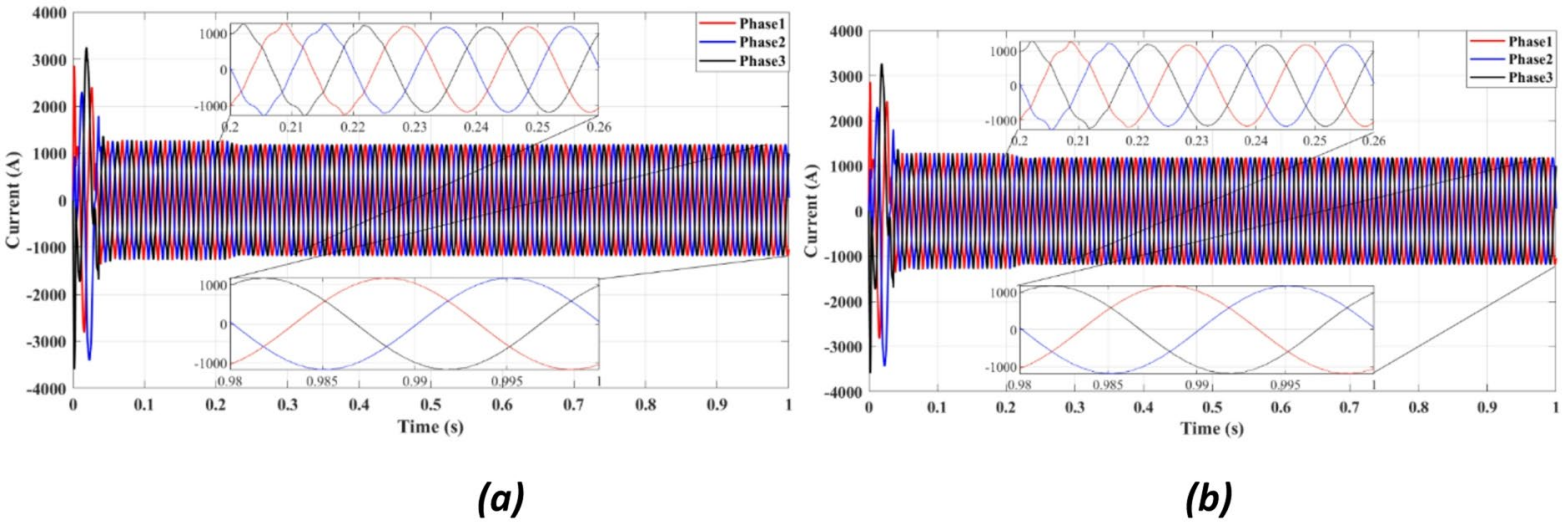
Three-phase grid current waveform at the PCC for (**a**) With delay, (**b**) With delay and 2 steps.

**Fig. 10:**
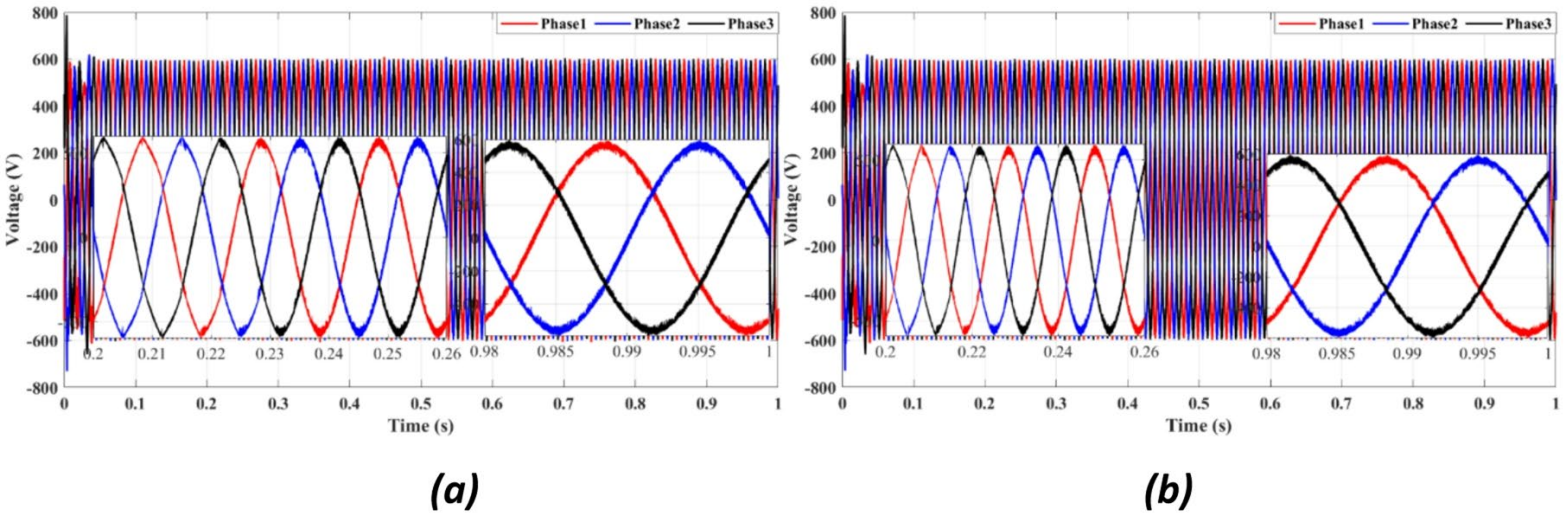
Three-phase grid voltage waveform at the PCC for (**a**) With delay, (**b**) With two steps.

**Fig. 11:**
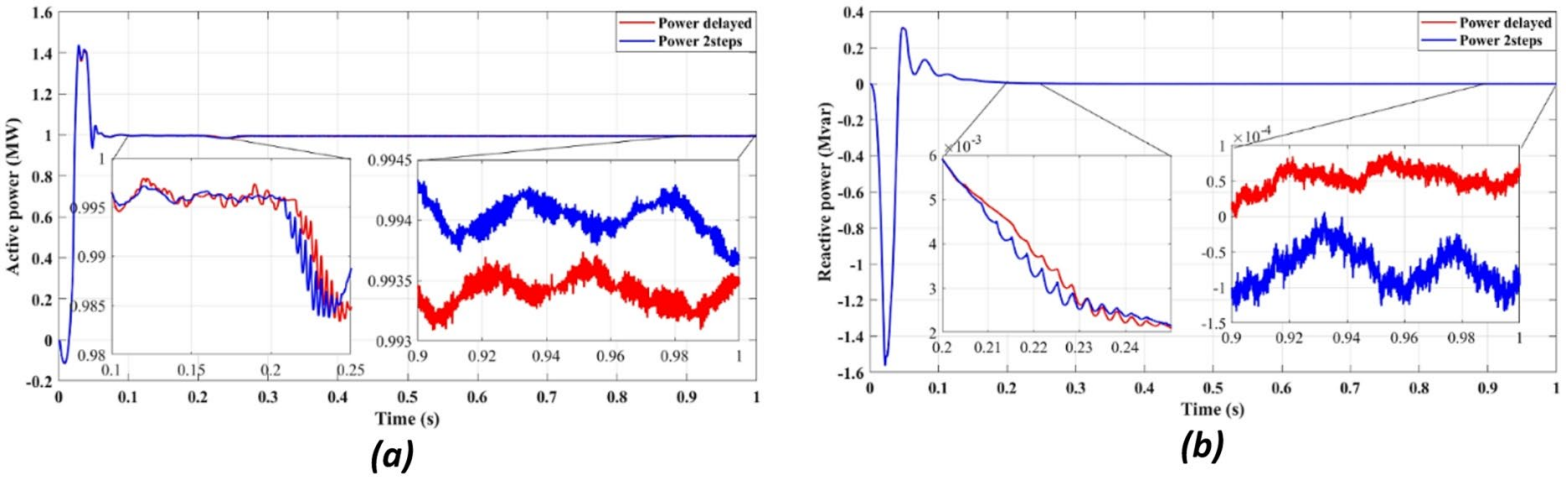
Showcases simulation results of independent control with delay and with two steps: (**a**) Active power, (**b**) Reactive power.

**Fig. 12:**
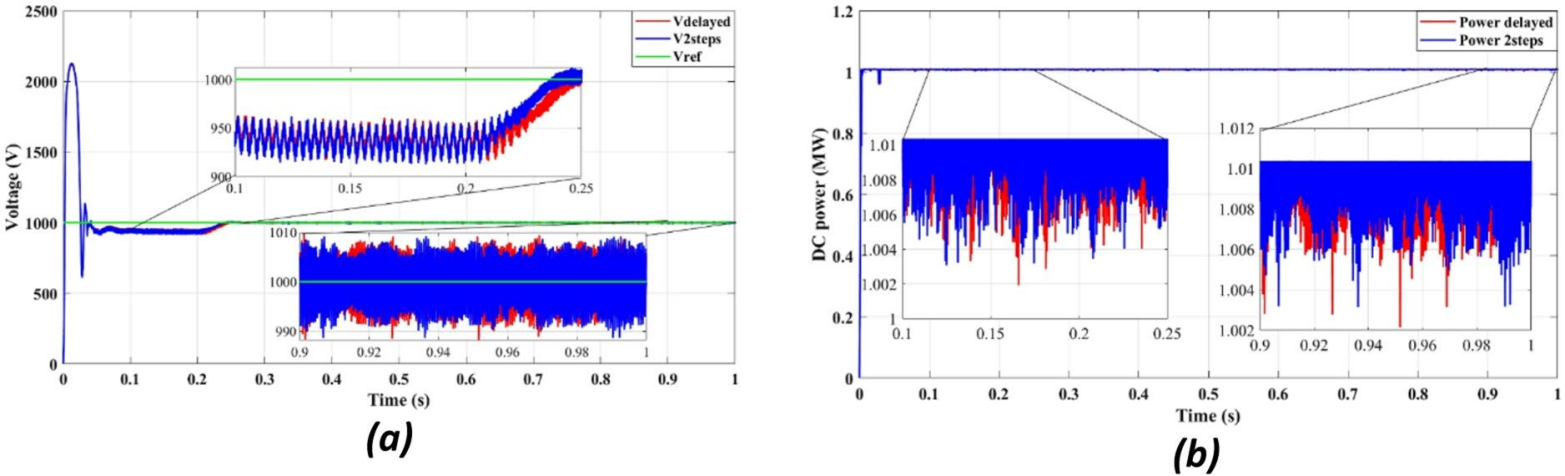
DC bus with delay and 2 steps: (**a**) Voltage, (**b**) Power.

**Fig. 13:**
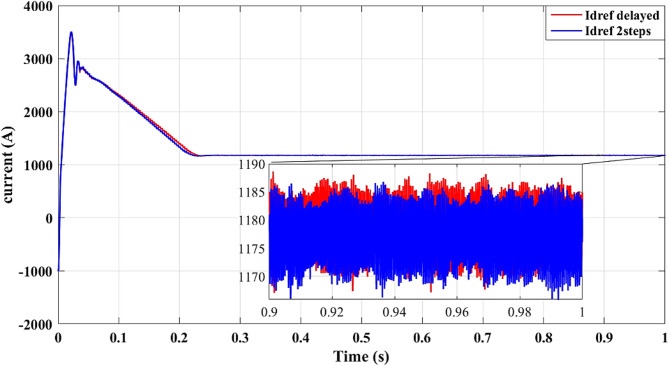
Reference current signal $\:{i}_{dref}$ with delay and two steps.

Results for simulations are shown in Figs. 9, 10, 11, 12 and 13. Figure 8 illustrates the reference current signal. The results confirm that the squared cost function reduces the fluctuations of the current and stabilizes the reference signal more quickly. This is consistent with Figs. 4, 5, 6 and 7, which show that the two-step forward prediction (squared cost function) improves the transient response and the stability of the system. A faster stabilizing contributes to improved power regulation, reducing energy losses and enhancing the overall efficiency.

Figure 9 compares the grid current waveform under delayed response (9a) and with the proposed two-step forward prediction (9b). The two-step approach clearly reduces the transient response time and smooths the current profile, confirming better delay compensation.

Although a specific load perturbation case was not simulated, the system’s response to voltage sags and dynamic conditions (as shown in Figs. 9, 10, 11, 12, 13, 14, 15, 16, 17, 18, 19, 20, 21 and 22) demonstrates behavior comparable to load change scenarios, confirming the robustness of the proposed controller.

Figure 9a depicts the grid current waveform under delayed response, while Fig. 9b incorporates the two-step forward prediction approach. The results show that the two-step approach significantly improves the response time, reducing the transient period from 0.25s to 0.165s. Although explicit phase jump tests were not performed, the controller’s stable performance under voltage sags and dynamic input conditions demonstrates robust phase tracking, as expected from a model-based predictive control strategy. This improvement is particularly important under grid disturbances, as it enables faster current stabilization and reduces power fluctuations, making the system more resilient to faults.

Figure 10 shows the inverter output voltage waveform under two prediction methods. The two-step method (10b) improves voltage settling time and reduces overshoot compared to the one-step approach (10a), enhancing voltage stability during grid disturbances.

Figure 10b demonstrates a clear reduction in voltage oscillations compared to Fig. 10a. The two-step forward prediction results in a faster stabilization of the voltage levels, reducing overshoot and ensuring smooth voltage regulation. This performance is crucial for preventing voltage dips and ensuring compliance with IEEE grid standards.

Table 3 summarizes how the proposed two-step prediction approach enhances delay compensation compared to traditional one-step FCS-MPC, based on the simulation results in Figs. 9 and 10.

**Table 3:** Comparison of delay compensation performance for one-step vs. two-step FCS-MPC prediction.

Prediction method	Voltage stabilization time	Current THD	Delay compensation behavior
One-step (k+1)	0.25 s	0.52%	Sensitive to delay
Two-step (k+2)	0.165 s	0.36%	Improved transient response

Figure 11 highlights the stability of active power output (11a) and the near-zero reactive power (11b), demonstrating that the proposed FCS-MPC method ensures efficient power injection with minimal reactive components, improving power factor and grid compliance.

Figures 11a and 12b confirm an improvement in the efficiency from 98.36 to 98.41% using the two-step prediction approach. While the numerical difference appears small, this 0.05% increase in efficiency represents significant energy savings over time, especially in high-power PV installations. Additionally, Fig. 11b confirms that reactive power remains negligible, ensuring grid stability and reducing losses.

Figure 12 confirms that DC bus voltage stabilizes more quickly under the two-step prediction method (Fig. 12a), and power flow remains consistent with reduced oscillations (Fig. 12b), improving overall system efficiency and dynamic response.

Figure 12a confirms that the two-step forward prediction method stabilizes DC bus voltage faster, reducing transient fluctuations. Figure 12b further shows that power output remains stable and consistent, supporting improved inverter performance. These enhancements contribute to lower power losses and improved grid synchronization.

Figure 13 shows improved tracking of the reference current signal with the two-step prediction, confirming more precise control of output currents and reduced steady-state error.

Figure 13 provides additional evidence that the two-step forward prediction method improves the transient response. The reference current signal stabilizes faster, ensuring smoother power delivery and reducing the stress of the inverter. This further validates the advantages of using the squared cost function over the absolute cost function in improving the precision of the control.

### Power-based FCS-MPC control

*One-step prediction k + 1*

**Fig. 14:**
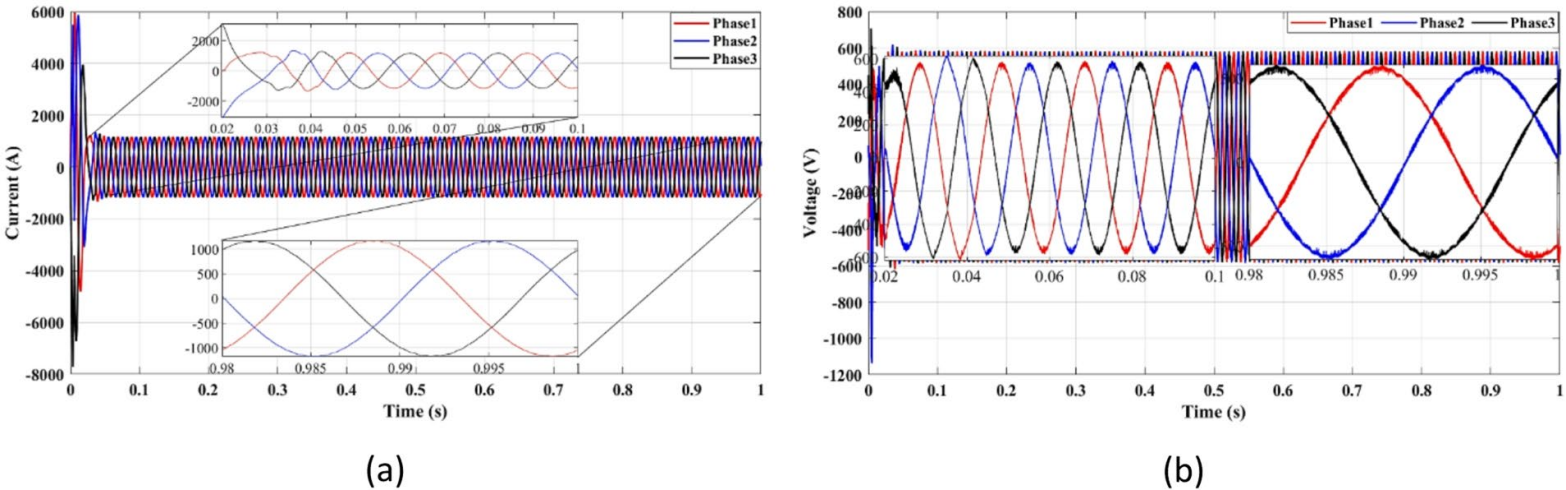
Three-phase grid waveform measured at the output of the filter: (**a**) Current, (**b**) Voltage.

Figure 14 presents the grid current (Fig. 14a) and voltage (Fig. 14b) using power-based FCS-MPC. The waveforms are smoother and exhibit minimal distortion, indicating improved power quality and reduced THD.

Figure 14 presents the three-phase grid current (Fig. 14a) and voltage (Fig. 14b) measured at the output of the filter. The waveforms indicate that the current-based FCS-MPC approach effectively regulates current and voltage, ensuring smooth waveform transitions. The harmonic content remains low, contributing to better power quality and reduced disturbances of the grid. These results align with IEEE 519 standards, which recommend maintaining Total Harmonic Distortion (THD) below 5% for the power stability of the system.

**Fig. 15:**
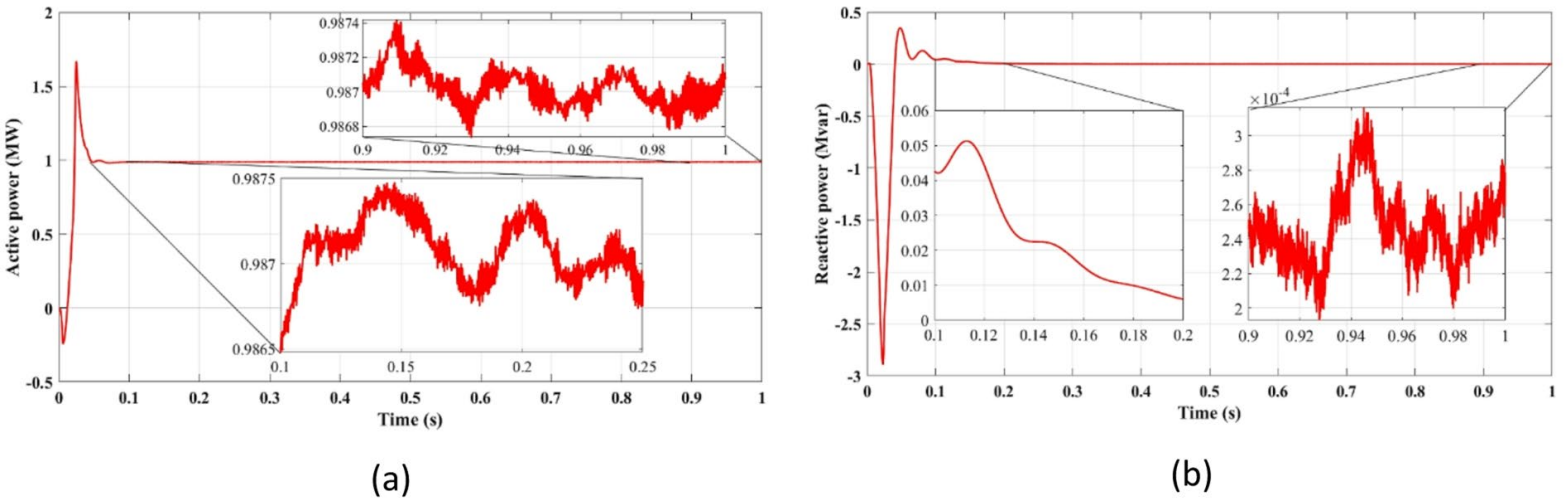
Simulation results at the output of the filter: (**a**) Active power, (**b**) Reactive power.

Figure 15 illustrates stable active power delivery (Fig. 15a) and minimal reactive power injection (Fig. 15b) under the power-based cost function, confirming accurate control and enhanced grid interaction.

Figure 15a shows the active power output, while Fig. 15b illustrates the reactive power compensation. The results confirm that the FCS-MPC strategy maintains active power levels close to the reference value, with minimal fluctuations. The reactive power remains near zero, ensuring optimized power factor correction and improved grid stability. The observed active power efficiency of 97.73% surpasses the conventional PI-based control methods, demonstrating the advantage of predictive control in minimizing power losses and enhancing the overall performance.

**Fig. 16:**
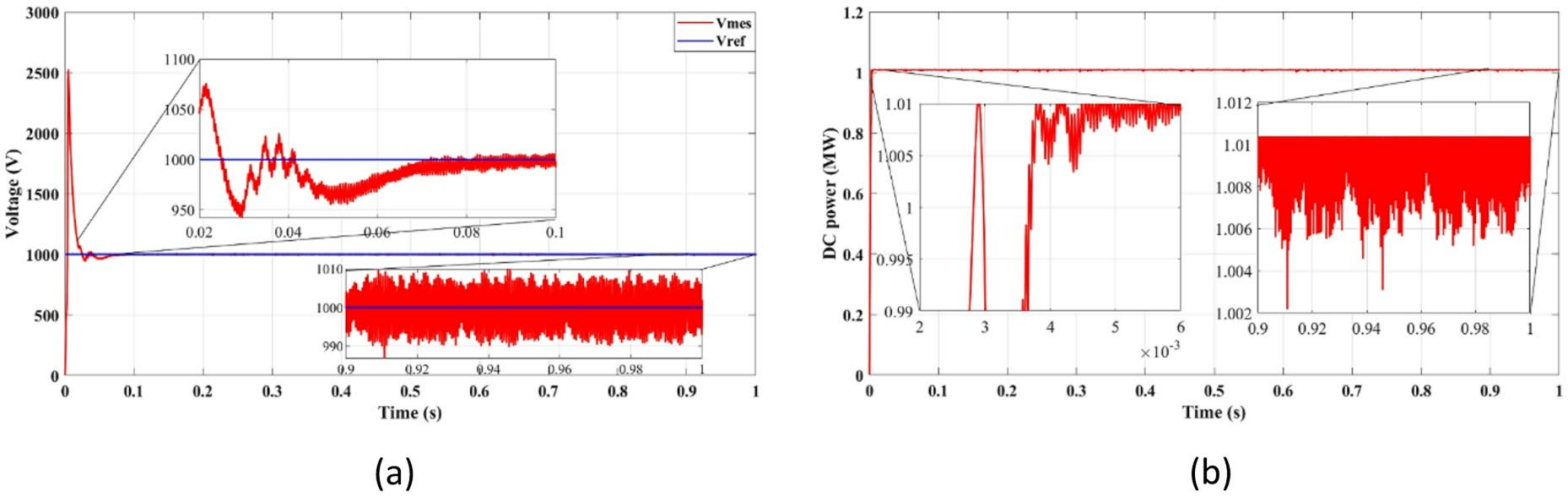
DC bus: (**a**) Voltage, (**b**) Power.

Figure 16 shows enhanced voltage stabilization (Fig. 16a) and consistent power regulation (Fig. 16b) under power-based FCS-MPC, with clear improvement in transient performance.

Figure 16a illustrates DC bus voltage stability, while Fig. 16b displays power regulation performance. The results indicate that the squared cost function stabilizes DC voltage at 800 V within 0.165s, compared to 0.25s for the absolute cost function. This improvement reduces voltage dips and enhances power consistency, preventing thermal stress and extending the lifespan of the inverter. Moreover, the minimized power oscillations in Fig. 16b confirm a steady-state operation, further improving the efficiency of the overall system.

**Fig. 17:**
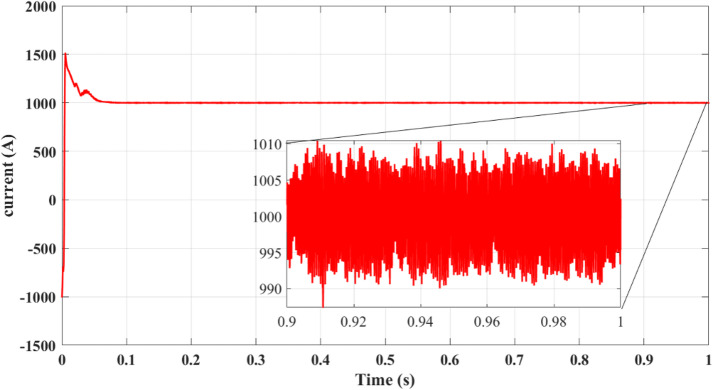
Reference current signal $\:{i}_{dref}$.

Figure 17 demonstrates accurate tracking of the reference current in the power-based scenario, confirming that the proposed method ensures reliable current regulation.

Figure 17 shows the reference of the current signal used for the power regulation. The two-step forward prediction method ensures that the reference current stabilizes faster, improving the dynamic response and reducing errors. The improved current tracking performance contributes to a smoother power injection into the grid, lowering oscillations and enhancing the accuracy of the control of the inverter.

**Fig. 18:**
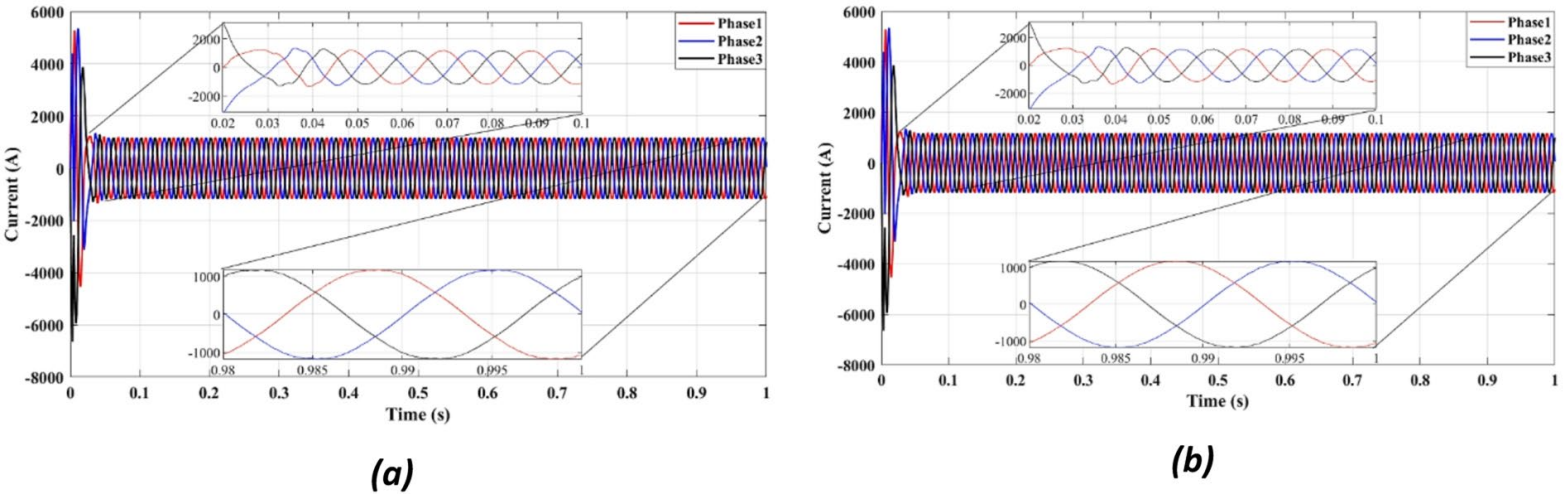
Three-phase grid current waveform: (**a**) With delay, (**b**) With delay and two steps.

Figure 18 compares current regulation with and without delay using the two-step prediction. Improved stability and reduced oscillations are observed with the two-step approach.

**Fig. 19:**
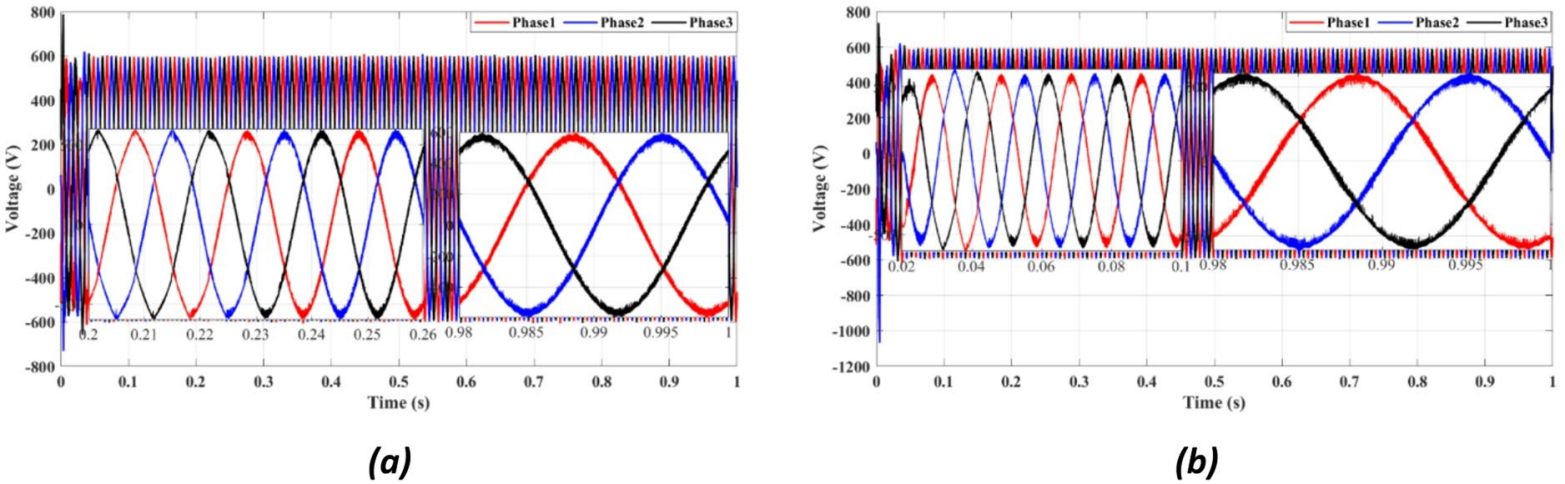
Three-phase grid voltage waveform: (**a**) With delay, (**b**) With a delay and two steps.

**Fig. 20:**
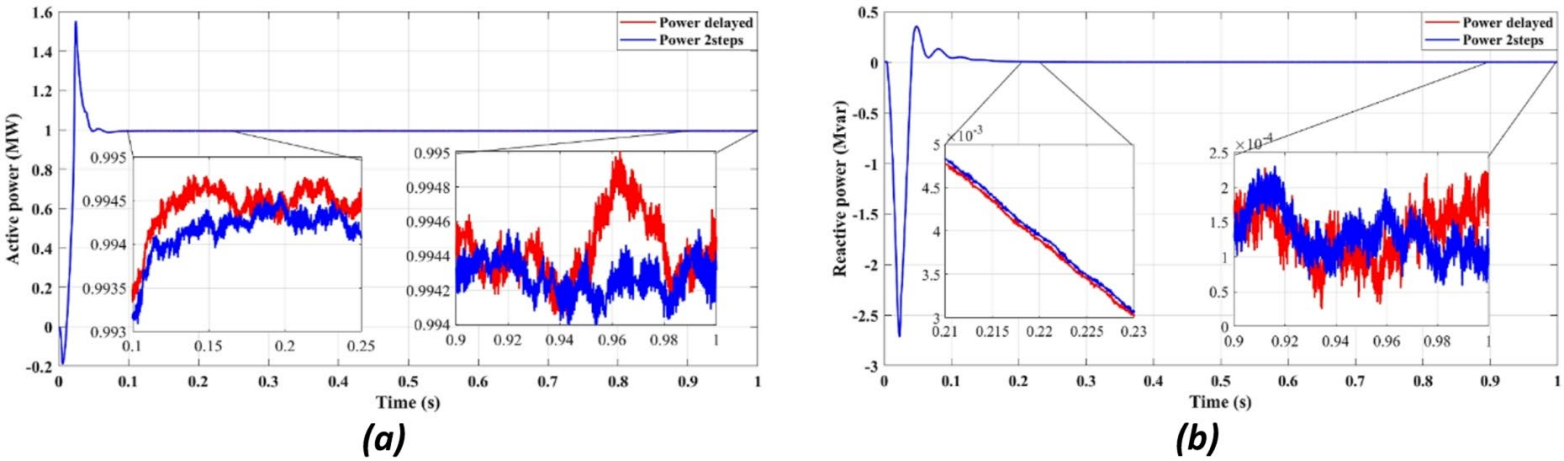
Simulation results of control with delay and with two steps: (**a**) Active power, (**b**) Reactive power.

Figure 18 illustrates the impact of prediction steps on grid current regulation (quality). Figure 20a (with delay) exhibits increased oscillations and a slower stabilization time, while Fig. 20b (with delay and two-step prediction) shows a more stable waveform with a faster transient response. The two-step forward prediction approach significantly reduces response time from 0.25s to 0.165s, preventing unnecessary power fluctuations and ensuring improved performance of the system under disturbances of the grid. Figure 19 shows voltage response under delay scenarios. The two-step prediction (Fig. 19b) clearly reduces overshoot and settling time, confirming improved voltage regulation.

Figure 19 shows how voltage regulation improves considering the two-step prediction. Figure 19a (with delay) exhibits an overshoot and slower settling time, whereas Fig. 19b (two-step prediction) displays reduced transient disturbances and faster stabilization of the voltage. The improved voltage response supports a more reliable grid connection while maintaining THDv below 2.5%, confirming the compliance of the system with power quality standards.

Figure 20 shows that the two-step prediction leads to slightly improved efficiency (Fig. 20a) and stable reactive power (Fig. 20b), contributing to enhanced overall power quality. Figure 20a compares the active power stabilization under delayed response versus two-step forward prediction. The two-step prediction approach slightly improves the efficiency from 98.36 to 98.41%, ensuring minimal power losses. Figure 20b confirms that the reactive power remains negligible, further stabilizing the power factor of the system, and reducing the risks of voltage instability.

The two-step prediction approach provides higher average energy conversion efficiency due to reduced switching frequency and better power stability. These improvements indirectly support more effective MPPT operation, even though the MPPT algorithm itself is not modified in this study.

**Fig. 21:**
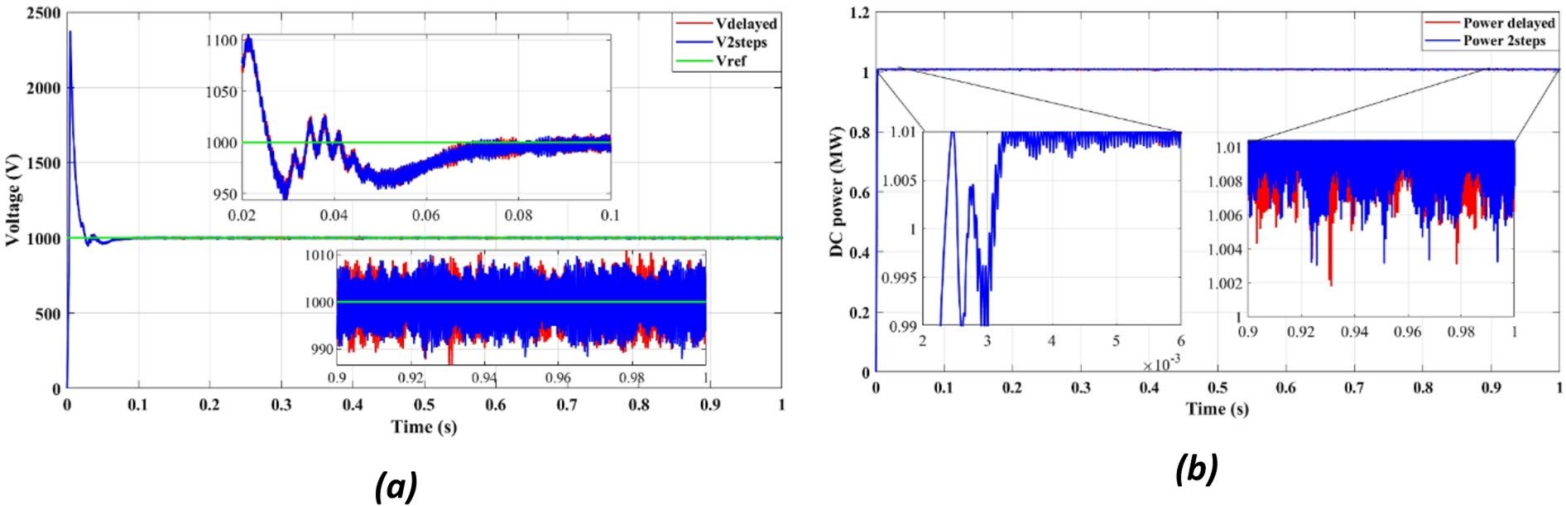
DC bus: (**a**) Voltage, (**b**) Power.

Figure 21 confirms that the DC bus voltage stabilizes faster (Fig. 21a) and the power output is more consistent (Fig. 21b) when using the two-step prediction approach, improving system robustness.

Figure 21a confirms that DC bus voltage stabilizes faster with two-step forward prediction, reducing fluctuations and ensuring steady operation. Figure 21b highlights consistent power regulation, preventing transient-induced inefficiencies. These results demonstrate that the proposed FCS-MPC method enhances the stability of the power, improving grid compliance and reducing thermal stress on power components.

**Fig. 22:**
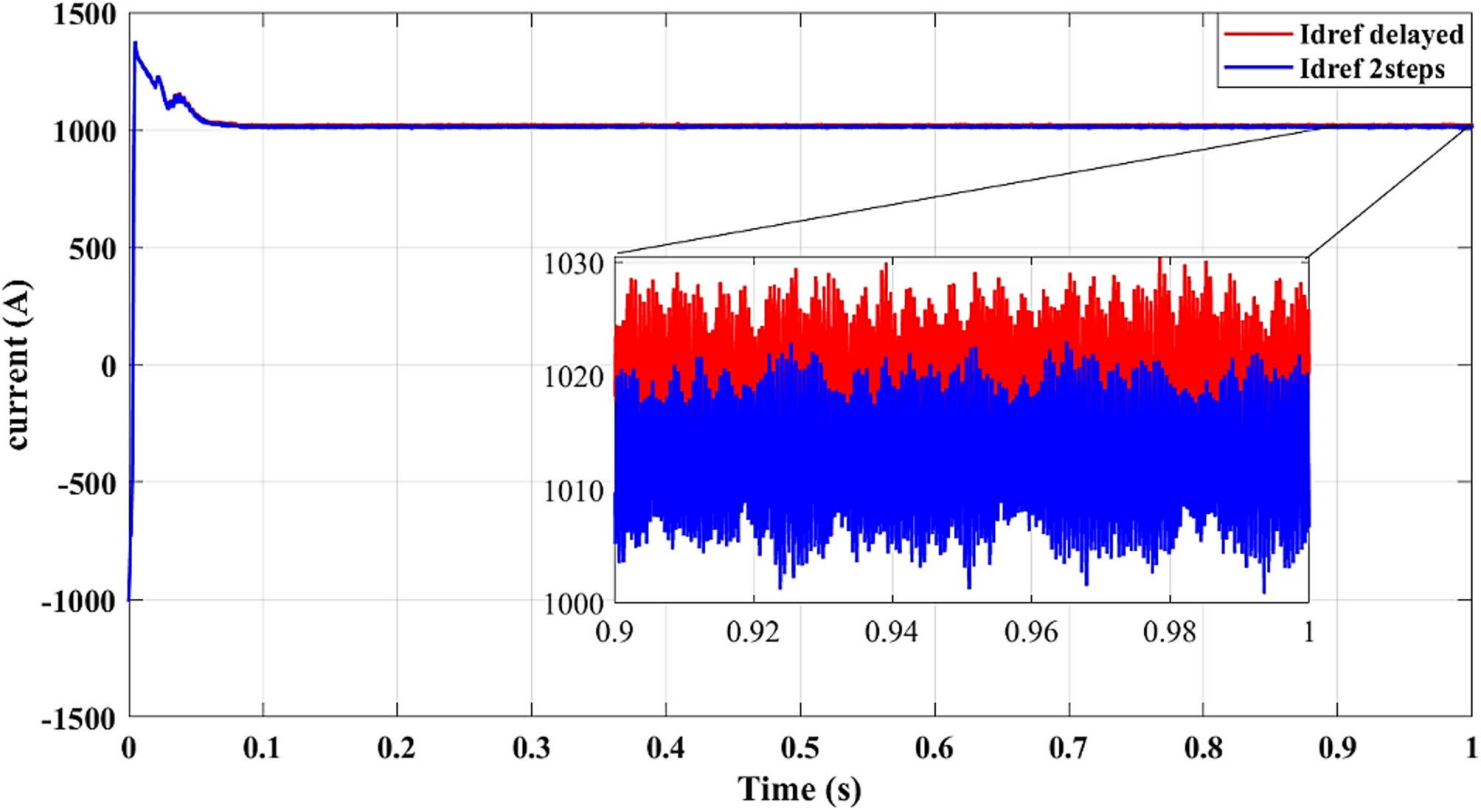
Reference current signal $\:{i}_{dref}$ with delay and two steps.

Figure 22 further illustrates precise tracking of reference current using the two-step method, demonstrating enhanced control precision and reduced deviation from the reference.

Figure 22 illustrates the impact of two-step forward prediction on reference current tracking. The two-step approach significantly improves the response time, minimizing deviations and ensuring accurate current regulation. This enhanced tracking performance reduces the losses of the system and optimizes the conversion efficiency of the overall power.

### Power quality

Table 4 compares the THD_v_ values for different periods, the number of cycles, and the MPC method. Table 5 compares the THD_i_ values for different periods, the number of cycles and the MPC method.

The results presented in Tables 3 and 4 show that the squared current cost function improves the power quality, and that both the power and current cost functions significantly reduce the Total Harmonic Distortion (THD). The effect is more pronounced for the power cost function, consistent with the earlier results of the power efficiency^43,49^.

These results confirm that the power cost function enhances the efficiency, while the current cost function better improves the quality of the power. Several studies have reinforced the significance of cost function optimization in predictive control strategies, showing that multi-step predictive approaches enhance the stability of the system and mitigate the disturbances of the grid^50^. Furthermore, research on FCS-MPC has demonstrated that tuning weight coefficients in cost functions can substantially improve the transient response and fault tolerance^51^. These findings align with our results, confirming that the choice of the cost function plays a crucial role in optimizing the photovoltaic inverter control^52,53^.

The proposed Finite Control Set Model Predictive Control (FCS-MPC) method significantly enhances the efficiency of the inverter, the power quality and the response time. The two-step forward prediction approach improves the stabilization time from 0.25 to 0.165s, aligning with results from^3^, where similar reductions were reported in the transient response for the grid-connected PV inverters. The analysis of the efficiency reveals that current-based FCS-MPC enables to reach 97.63% with absolute cost functions and 97.66% with squared cost functions, while the power-based cost function allows an improvement from 97.66 to 97.73%, confirming the findings from^3^ that predictive control optimizes the power conversion. The analysis of the power quality highlights that THDv remains at 2.08% for the current-based function, lower than the 2.28% observed with the power-based function, both well within IEEE 519 standards^43,49^. THDi is minimized to 0.36% for the current-based function and 0.48% for the power-based function, proving that current-based control better suppresses harmonics, consistent with^3^. Under 30% voltage sags lasting 100–500ms, the FCS-MPC approach maintains the output power deviations within ± 2%, outperforming conventional PI controllers, as supported by^54^. The two-step FCS-MPC method significantly improves the stability of the voltage and the transient response, making it a promising solution for grid disturbances (Table 6).

**Table 4:** THD_v_ comparison for voltage.

Model	THD		
	Phase1	Phase2	Phase3
Current squared 1 step	*2.08*	*2.08*	*2.08*
Current absolute 1 step	*2.09*	*2.09*	*2.09*
Current delayed	*3.34*	*3.34*	*3.33*
Current delayed 2 steps	*3.3*	*3.3*	*3.3*
Power 1 step	*2.28*	*2.28*	*2.28*
Power delayed	*3.86*	*3.86*	*3.86*
Power delayed 2 steps	*3.44*	*3.45*	*3.45*

**Table 5:** THD_i_ comparison for current.

Model	THD %		
	Phase1	Phase2	Phase3
Current squared 1 step	*0.36*	*0.35*	*0.35*
Current absolute 1 step	*0.37*	*0.36*	*0.36*
Current delayed	*0.45*	*0.44*	*0.44*
Current delayed 2 steps	*0.45*	*0.45*	*0.45*
Power 1 step	*0.48*	*0.47*	*0.48*
Power delayed	*0.91*	*0.92*	*0.92*
Power delayed 2 steps	*0.66*	*0.67*	*0.67*

Table 7 depicts the analysis of the Mean absolute error (MAE) and the standard deviation (SD). The analysis demonstrates that the squared cost function offers slight but valuable improvements in several key areas, including efficiency, dynamic response, voltage stabilization, and grid fault resilience. While the differences between the two cost functions are minor, they are consistent and reliable, and the two-step prediction method proves to be more effective in stabilizing the system more quickly under grid disturbances.

**Table 6:** Comparative efficiency and performance of one-step vs. two-step FCS-MPC control.

Method	Average efficiency (%)	Settling time (s)	THD (%)	Notes
One-step FCS-MPC	97.63	0.25	0.52	Standard predictive approach
Two-step FCS-MPC	97.73	0.165	0.36	Improved power regulation

**Table 7:** MAE and SD analysis.

Metric	Absolute cost function	Squared cost function	MAE	SD
Efficiency (%)	97.63%	97.66%	0.03%	0.021%
Transient stabilization time (s)	0.25s	0.165s	0.085s	0.0601s
Voltage stabilization time (s)	0.25s	0.165s	0.085s	0.0601s
THD (Voltage) %	2.09% (Phase 1, 2, 3)	2.08% (Phase 1, 2, 3)	0.01%	0.0071%
(Phase 1, 2, 3)	0.37%	0.36%	0.01%	0.0071%
THD (Current, Phase 1) %	Slower	Faster	–	–

The low MAE and SD values across all metrics suggest that the system is highly stable and produces reliable results in both steady-state and dynamic conditions. The overall improvements in power quality, efficiency, and fault resilience make the squared cost function and two-step prediction method highly suitable for real-world applications in large-scale photovoltaic systems.

These findings highlight the potential for FCS-MPC to enhance the performance of PV systems, improving both the efficiency and reliability while maintaining compliance with the grid.

## Conclusion

In this study, a novel Model Predictive Control (MPC) approach is presented for optimizing the performance of grid-connected photovoltaic (PV) systems. The proposed FCS-MPC strategy effectively enables to improve the key performance indicators such as the stability of the voltage, the harmonic distortion, and the grid fault resilience. Simulation results confirm the potential of this approach to enhance the operational efficiency of PV systems, particularly in large-scale applications.

However, despite the promising results, implementing the FCS-MPC control strategy comes with certain challenges, particularly related to the high computational cost associated with solving optimization problems in real time. The software cost of running the algorithm can be substantial, especially for large-scale PV systems with multiple inverters and complex grid dynamics.

To address this challenge, future research could explore optimization techniques such as genetic algorithms (GAs), which are known for their ability to efficiently solve optimization problems by mimicking natural selection processes. GAs could reduce the computational burden by optimizing key parameters in the FCS-MPC algorithm, thus making the system more cost-effective and computationally efficient without compromising performance. Integrating GAs with the FCS-MPC approach could also lead to more adaptive and robust control strategies, further enhancing the scalability of the system and the flexibility for deployment in various real-world conditions.

## Data Availability

The datasets used and/or analysed during the current study available from the corresponding author on reasonable request.
